# SGLT2 Inhibitors in Autosomal Dominant Polycystic Kidney Disease: A Systematic Review and Meta-Analysis

**DOI:** 10.7759/cureus.107653

**Published:** 2026-04-24

**Authors:** Ayoub Tantush, Bahaeddin Ben Hamida, Malek Sagher, Nadia Afsheen, Motasem Sager, Allaeddin Abusbaeh, Marwan Tantoush, Ali Abusbaeh, Abdelrahman Najaim, Mohammed Alhamadi, Mohamed Hesham Gamal, Mohamad M Alemam, Mahmoud M Elhady

**Affiliations:** 1 General Medicine, University Hospitals Birmingham NHS Foundation Trust, Birmingham, GBR; 2 General Medicine, Heartlands Hospital, University Hospitals Birmingham NHS Foundation Trust, Birmingham, GBR; 3 Internal Medicine, Heartlands Hospital, University Hospitals Birmingham NHS Foundation Trust, Birmingham, GBR; 4 Geriatrics, Heartlands Hospital, University Hospitals Birmingham NHS Foundation Trust, Birmingham, GBR; 5 Internal Medicine, University Hospitals Birmingham NHS Foundation Trust, Birmingham, GBR; 6 General Practice, Calderdale and Huddersfield NHS Foundation Trust, Huddersfield, GBR; 7 Internal Medicine, Russell’s Hall Hospital, The Dudley Group NHS Foundation Trust, Dudley, GBR; 8 General Medicine, Queen Elizabeth Hospital, University Hospitals Birmingham NHS Foundation Trust, Birmingham, GBR; 9 Pharmacology and Therapeutics, Faculty of Pharmacy, Tanta University, Tanta, EGY; 10 General Practice, Faculty of Medicine, Alexandria University, Alexandria, EGY; 11 Orthopedics, Faculty of Medicine, Benha University, Qalubiya, EGY

**Keywords:** autosomal dominant polycystic kidney disease, egfr, meta-analysis, renoprotection, sglt2 inhibitors

## Abstract

Autosomal dominant polycystic kidney disease (ADPKD) is the most common inherited kidney disorder, with tolvaptan remaining the primary disease-modifying therapy. SGLT2 inhibitors have demonstrated robust renoprotection across the chronic kidney disease (CKD) spectrum, yet patients with ADPKD have been systematically excluded from pivotal trials due to concerns over vasopressin-mediated cystogenesis. This systematic review and meta-analysis aimed to pool available evidence on SGLT2 inhibitor use in patients with ADPKD. This systematic review and meta-analysis were registered with PROSPERO (CRD420261324155) and conducted in accordance with Preferred Reporting Items for Systematic Reviews and Meta‑Analyses (PRISMA) guidelines. Five databases were searched through February 2026 for studies including ADPKD patients receiving any SGLT2 inhibitor. Quality was assessed using the Risk of Bias 2 (RoB2), Newcastle-Ottawa Scale, and Joanna Briggs Institute (JBI) tools, depending on the study design. Eight studies encompassing 3,180 patients were included, comprising one randomized controlled trial (RCT), one target trial emulation study, and six retrospective observational studies. In comparative analyses, SGLT2 inhibitor use was associated with a statistically significant attenuation of estimated glomerular filtration rate (eGFR) decline (pooled mean difference {MD}: 1.344 mL/min/1.73 m²/year; 95% CI: 0.836-1.852). A significant hemoglobin increase was observed in both comparative (MD: 0.66 g/dL) and single-arm analyses (MD: 0.55 g/dL). Subgroup analyses suggested a greater eGFR benefit in non-diabetic patients, though differences were not statistically significant. Our findings suggest that SGLT2 inhibitors may attenuate eGFR decline and improve hemoglobin in ADPKD, with the initial eGFR dip representing a class effect rather than harm, supporting their prospective evaluation in dedicated randomized trials. These findings should be interpretedwith cuchion the predominantly observational study designs, heterogeneous patient populations

## Introduction and background

Autosomal dominant polycystic kidney disease (ADPKD) is the most common inherited kidney disorder worldwide. It is characterized by progressive bilateral renal cyst development that inexorably leads to end-stage kidney disease (ESKD) in approximately 50% of affected individuals by the sixth decade of life [1]. Current management centers on renin-angiotensin system (RAS) inhibition and tolvaptan, a vasopressin V2-receptor antagonist, which has been demonstrated in landmark randomized controlled trials to slow total kidney volume (TKV) growth and attenuate decline in kidney function [2,3].

Sodium-glucose cotransporter-2 inhibitors (SGLT2i) have demonstrated robust renoprotective effects across the spectrum of chronic kidney disease (CKD) in large-scale trials, including DAPA-CKD and EMPA-KIDNEY [4,5]. Nevertheless, patients with ADPKD were systematically excluded from these trials due to concerns that SGLT2i-induced elevation of vasopressin levels could theoretically accelerate cystogenesis and hasten disease progression based on limited mechanistic evidence; consequently, the 2025 KDIGO clinical practice guidelines for ADPKD do not endorse SGLT2i use due to insufficient safety data [3,6]. Beyond SGLT2i, the therapeutic landscape for glucose-lowering agents continues to expand rapidly, with novel agents demonstrating increasingly favorable metabolic and renal profiles in patients with type 2 diabetes and obesity [7,8].

Growing evidence has since begun to address this knowledge gap. Observational studies and case series have reported variable outcomes with dapagliflozin in ADPKD, with some reporting attenuation of estimated glomerular filtration rate (eGFR) decline and others raising concern regarding TKV growth [9]. This discordance likely reflects the dissociation between hemodynamic eGFR effects and true structural disease modification in ADPKD, underscoring the need for concurrent height-adjusted TKV (htTKV) assessment in future studies [9]. More recently, the first open-label, randomized, controlled, crossover trial demonstrated significant attenuation of both eGFR decline and TKV growth with the addition of dapagliflozin to tolvaptan background therapy [10].

Given this emerging evidence, this systematic review and meta-analysis aimed to synthesize available evidence on the efficacy and safety of SGLT2 inhibitors in patients with ADPKD, with particular focus on eGFR slope attenuation, initial eGFR dip, and hemoglobin changes, to inform clinical decision-making pending dedicated randomized trial data.

## Review

Methods

Registration and Reporting

This systematic review and meta-analysis were conducted in accordance with the Cochrane Handbook for Systematic Reviews of Interventions and reported following the Preferred Reporting Items for Systematic Reviews and Meta-Analyses (PRISMA) guidelines [11,12]. Our protocol was registered on PROSPERO with the number CRD420261324155.

Search Strategy

We systematically searched PubMed, Scopus, Cochrane, Web of Science, and Embase for relevant studies published up to February 2026. The search combined terms related to the population and intervention, including: ("autosomal dominant polycystic kidney disease" OR "ADPKD" OR "polycystic kidney") AND ("SGLT2 inhibitor" OR "sodium-glucose cotransporter-2" OR "dapagliflozin" OR "empagliflozin" OR "canagliflozin"). No language restrictions were applied, and reference lists of included studies were manually screened for additional eligible records. The full search strategy is presented in the Appendices.

Study Selection and Eligibility Criteria

Two independent reviewers screened titles, abstracts, and full texts. Studies were included if they met the following criteria: (1) population: patients with a confirmed or clinically established diagnosis of ADPKD; (2) intervention: any SGLT2 inhibitor; (3) comparator: standard care, tolvaptan monotherapy, or another active comparator; and (4) outcomes: at least one of the pre-specified outcomes of interest. Studies were excluded if they were animal models, non-human experimental studies, review articles without original data, or if they did not report extractable quantitative outcomes.

Data Extraction

Data were extracted independently by two reviewers using predesigned standardized Excel sheets into two main categories: (1) study summary data, including study ID, country, study design, sample size, intervention details, follow-up duration, and primary outcomes; and (2) baseline characteristics, including mean age, sex distribution, mean eGFR at baseline, presence of diabetes mellitus (DM), hypertension (HTN), concurrent medications (tolvaptan, RAS inhibitors), and Mayo imaging classification where available. Discrepancies were resolved by discussions with a third author.

Quality Assessment

The methodological quality of the included studies was assessed using two validated tools, based on study design. The Cochrane Risk of Bias Tool version 2 (RoB2) was applied to the randomized controlled trial, evaluating five domains: randomization process, deviations from intended interventions, missing outcome data, measurement of outcomes, and selection of reported results [13]. Each domain was rated as low risk, some concerns, or high risk. The Newcastle-Ottawa Scale (NOS) was applied to observational studies to assess selection, comparability, and outcome domains, with scores ranging from 0 to 9 stars [14]. For case series studies, the JBI Critical Appraisal Checklist for Case Series was applied, evaluating 10 criteria spanning inclusion criteria clarity, standardized condition measurement, participant demographics and clinical reporting, outcome reporting, and statistical appropriateness. Each criterion was rated as yes, no, unclear, or not applicable. All appraisals were performed independently by two reviewers, with disagreements resolved by consensus [15].

Outcome Measures

The primary outcome was the change in eGFR slope (ΔeGFR slope, mL/min/1.73 m²/year) (by the calculation of pre- and post-values) following initiation of an SGLT2 inhibitor compared with control. Secondary outcomes included: the magnitude of the initial eGFR dip after SGLT2 inhibitor initiation (expressed as percentage change from baseline), and the change in height-adjusted total kidney volume (htTKV, %/year). Subgroup analyses were performed by diabetes mellitus and hypertension status to explore potential sources of heterogeneity in the eGFR slope response.

Statistical Analysis

Statistical analyses were performed using the meta package in R (R Foundation for Statistical Computing, Vienna, Austria, https://www.R-project.org), with a significance threshold set at p < 0.05. For continuous outcomes, the mean difference (MD) and 95% confidence interval (CI) were calculated. For single-arm outcomes without a comparator group, the mean raw (MRAW) with 95% CI was pooled. Heterogeneity was assessed using the I² statistic and the chi-square test; data were considered heterogeneous when I² > 50% and chi-square p < 0.1. A fixed-effects model was applied to homogeneous data, and a random-effects model to heterogeneous data. Sensitivity analyses were performed using a leave-one-out approach to assess the stability of the pooled estimates and to identify potentially influential studies [16].

Results

Study Selection and Included Studies

**Figure 1:**
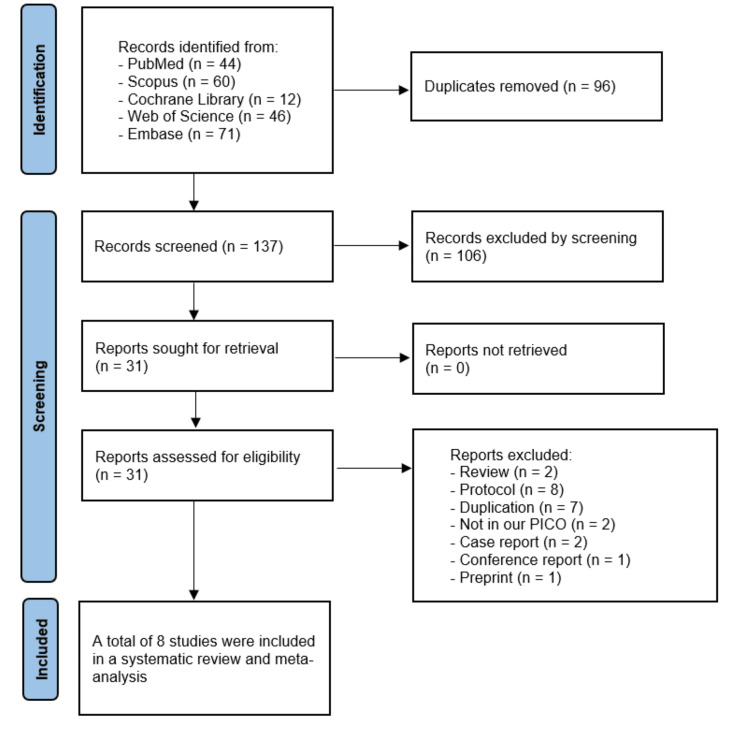
PRISMA flow diagram Stepwise representation of the study selection process from database searches to final inclusion of eight studies. PRISMA = Preferred Reporting Items for Systematic Reviews and Meta‑Analyses

The systematic search identified records across PubMed, Scopus, Web of Science, Cochrane Library, and Embase. After removing duplicates (96 records) and screening titles and abstracts (106 records), a full-text review was performed on 31 eligible records. The final included studies were eight for the systematic review and meta-analysis [10,17-23]. The full details are shown in the PRISMA flow diagram (Figure 1).

Baseline Characteristics and Summary

A total of eight studies were included, comprising 3,180 patients across Japan, the United States, and Canada. Study designs included one open-label randomized controlled crossover trial, one target trial emulation study, and six retrospective observational studies or case series, with follow-up durations ranging from approximately three months to seven years. The predominant SGLT2 inhibitor used was dapagliflozin 10 mg/day, with some studies including empagliflozin or mixed SGLT2 inhibitor regimens. Comparator groups varied across studies, including tolvaptan monotherapy, DPP4 inhibitors, GLP-1 receptor agonists, or no comparator in single-arm studies. Sample sizes ranged from six to 2,640 matched pairs. Patients had a mean age ranging from the late thirties to the mid-seventies, with diabetes mellitus and hypertension variably present across studies. All patients in the tolvaptan combination studies were on background RAS inhibitor therapy. Mayo imaging classification ranged from 1B to 1E, where reported. The full details of baseline patient data and study characteristics are presented in Tables 1, 2.

**Table 1:** Baseline characteristics [10,17–23] ACEi = Angiotensin-converting enzyme inhibitor; ARB = Angiotensin receptor blocker; BMI = Body mass index; DBP = Diastolic blood pressure; DPP4i = Dipeptidyl peptidase-4 inhibitor; eGFR = Estimated glomerular filtration rate; htTKV = Height-adjusted total kidney volume; IQR = Interquartile range; NR = Not reported; PSM = Propensity score matching; RAS = Renin-angiotensin system; SBP = Systolic blood pressure; SD = Standard deviation; SGLT2i = Sodium-glucose cotransporter-2 inhibitor; T2DM = Type 2 diabetes mellitus; TKV = Total kidney volume; UPCR = Urine protein-to-creatinine ratio

Study ID	Study arm	Patients number	Age (years)	Male, n (%)	BMI (kg/m²)	eGFR (mL/min/1.73m²)	TKV (mL)	htTKV (mL/m)	SBP (mmHg)	DBP (mmHg)	Diabetes, n (%)	Hypertension, n (%)	Tolvaptan use, n (%)	RAS inhibitor, n (%)	Hemoglobin (g/dL)	Uric acid (mg/dL)	Serum albumin (g/dL)	UPCR (mg/gCr)
Cau 2025 [17]	SGLT2i	17	66.1 (mean)	10 (58%)	NR	40.9 (mean)	NR	NR (8/17 had TKV data)	NR	NR	10/15 (67%)	NR	2/13 (15%)	13/13 (100%)	NR	NR	NR	NR
Morioka 2023 [19]	Dapagliflozin	20	51 (46-57)	9 (45%)	23.0 (20.9-24.8)	47.9 (39.7-56.9)	967 (689-1168)	599 (423-707)	135 (129-142)	86 (79-94)	1 (5%)	14 (70%)	11 (55%)	11 (55%)	13.1 (12.2-14.4)	NR	4.4 (4.2-4.5)	NR
Nakajima 2025 [20]	Tolvaptan	24	50 ± 12	11 (45.8%)	22.3 ± 2.9	52.4 ± 22.1	1491 ± 800	NR	NR	NR	0 (0%)	20 (83.3%)	24 (100%)	16 (66.7%)	12.5 ± 1.0	5.4 ± 1.3	4.0 ± 0.3	128.9 ± 157.6
	Dapagliflozin + Tolvaptan	24	47 ± 8	11 (45.8%)	21.7 ± 2.5	44.8 ± 19.2	1575 ± 970	NR	NR	NR	0 (0%)	15 (62.5%)	24 (100%)	15 (62.5%)	13.1 ± 1.6	5.9 ± 1.0	4.2 ± 0.4	144.3 ± 182.8
Nishida 2025 [21]	Dapagliflozin + Tolvaptan	4	55 (39-74)	1 (25%)	27.6 (22.2-39)	37.6 (27.3-58.7)	2396 (1001-5368)	1554 (595-3508)	124 (102-148)	79 (71-86)	0 (0%)	4 (100%)	3/4 (75%)	4 (100%)	11.7 (10.3-13)	5.9 (4.1-6.8)	NR	NR
	Tolvaptan	2	62 (50-74)	1 (50%)	29.1 (1 case)	17.3 (16.1-18.4)	795 (651-938)	486 (399-572)	138 (136-139)	83 (80-86)	0 (0%)	2 (100%)	2 (100%)	2 (100%)	10.8 (10.4-11.2)	3.9 (3.8-4.0)	NR	NR
Eswarappa 2025 [18]	SGLT2i (all)	348	68 ± 11	325 (93%)	NR	53 (16-127)	NR	NR	NR	NR	217 (62%)	329 (95%)	1 (0.3%)	227 (65%)	NR	NR	NR	61 (0-4219)
	SGLT2i (T2DM)	217	67 ± 10	201 (93%)	32.9 ± 5.9	56 (19-127)	NR	NR	NR	NR	217 (100%)	205 (94%)	0 (0%)	144 (66%)	NR	NR	NR	56 (0-1974)
	DPP4i	198	67 ± 12	184 (93%)	32.8 ± 6.6	53 (18-177)	NR	NR	NR	NR	198 (100%)	181 (91%)	0 (0%)	118 (60%)	NR	NR	NR	46 (0-2800)
Uchiyama 2025 [10]	All (crossover)	27	49.7 ± 12.1	14 (52%)	23.3 ± 2.9	61.4 ± 25.3	1400 (935-1480)	838 (550-905)	132.7 ± 12.5	84.2 ± 7.9	0 (0%)	22 (81%)	27 (100%)	18 (67%)	13.6 ± 1.4	6.19 ± 1.37	4.29 ± 0.29	NR
Yen 2025 [22]	SGLT2i (PSM)	2640	64.6 ± 10.9	1460 (55%)	≥30: 57%	67.9 ± 24.9	NR	NR	>130: 63%	>80: 56%	2640 (100%)	2060 (78%)	<11	ACEi 854; ARB 813	NR	NR	NR	51.1 ± 67.3
	Non-SGLT2i (PSM)	2640	64.6 ± 11.3	1434 (54%)	≥30: 59%	65.5 ± 26.1	NR	NR	>130: 61%	>80: 54%	2640 (100%)	2039 (77%)	<11	ACEi 843; ARB 828	NR	NR	NR	49.6 ± 64.9
Yoshimoto 2024 [23]	All (single-arm)	7	52 (37-67)	5 (71%)	NR	26.9 (22.6-43.1)	NR	1444 (517-2320)	NR	NR	0 (0%)	7 (100%)	4 (57%)	6 (86%)	13.6 ± 1.4	6.19 ± 1.37	NR	0.04-0.40 g/gCr

**Table 2:** Summary of the included studies [10,17–23] ADPKD = Autosomal dominant polycystic kidney disease; AKI = Acute kidney injury; BMI = Body mass index; BP = Blood pressure; CKD = Chronic kidney disease; DPP4i = Dipeptidyl peptidase-4 inhibitor; eGFR = Estimated glomerular filtration rate; GLP-1RA = Glucagon-like peptide-1 receptor agonist; GU = Genitourinary; Hb = Hemoglobin; Hct = Hematocrit; HF = Heart failure; htTKV = Height-adjusted total kidney volume; IHD = Ischemic heart disease; ITS = Interrupted time series; RCT = Randomized controlled trial; SGLT2i = Sodium-glucose cotransporter-2 inhibitor; T1DM = Type 1 diabetes mellitus; T2DM = Type 2 diabetes mellitus; TKV = Total kidney volume; UA = Uric acid; Uosm = Urine osmolality; UPCR = Urine protein-to-creatinine ratio

Study ID	Study design	Country	Intervention	Comparator	Sample size (n)	Follow-up duration	Inclusion criteria	Primary endpoint	Secondary endpoints
Cau 2025 [17]	Retrospective cohort study	British Columbia, Canada	(empagliflozin (9/17, 53%) and dapagliflozin 8/17 (47%))	None	17	Median 20.89 months	Adults ≥18 years with CKD and primary diagnosis of ADPKD	Acute kidney injury (AKI)	eGFR slope pre/post SGLT2i; eGFR dip magnitude; GU infections requiring hospital/ER/outpatient treatment
Morioka 2023 [19]	Retrospective case series	Japan	Dapagliflozin 10 mg/day	None	20	102 ± 20 days (range 70-156)	ADPKD patients; eGFR 25-75 mL/min/1.73m²	Changes in htTKV and eGFR	Annual htTKV change rate; changes in clinical parameters; correlation between htTKV change and urinary phosphate
Nakajima 2025 [20]	Retrospective cohort study	Japan	Dapagliflozin + Tolvaptan	Tolvaptan alone	48	Mean 649 ± 363 days	ADPKD patients receiving tolvaptan	Chronic eGFR slope	Initial eGFR dip; TKV changes; Hb, Hct, UA changes; within-group ITS analysis
Nishida 2025 [21]	Retrospective case series	Japan	Dapagliflozin + Tolvaptan	Tolvaptan alone	6	More than 2 years (DAPA group: 854 days)	ADPKD patients at their institution receiving tolvaptan ± dapagliflozin	eGFR decline rate	Changes in htTKV; changes in BP, BMI, UPCR, Uosm, Hb, UA
Eswarappa 2025 [18]	Retrospective cohort study	USA	SGLT2i	DPP4i (for T2DM)	415	1 year post; 1 year pre	Age ≥18; ADPKD ; SGLT2i/DPP4i	Mean eGFR change per 90 days	eGFR slope pre/post; initial dip; SGLT2i vs DPP4i comparison
Uchiyama 2025 [10]	Open-label RCT	Japan	Dapagliflozin + tolvaptan	Tolvaptan alone	27	6 mo/period (12 mo total)	Age >20; ADPKD; tolvaptan >60 mg/d for >3 mo; eGFR >25; no diabetes	eGFR slope (1-6 months)	TKV change %; weight; vasopressin; BP; urine parameters
Yen 2025 [22]	Target trial emulation	USA	SGLT2i	Non-SGLT2i; DPP4i; GLP-1RA	2640 pairs	Up to 7 years (2015-2022)	PKD + T2DM; age ≥20; excluded T1DM	Dialysis; IHD; HF; mortality; AKI	Progression to macroalbuminuria
Yoshimoto 2024 [23]	Retrospective cohort study	Japan	Dapagliflozin	None	7	Median 20 months	ADPKD; dapagliflozin	Change in eGFR slope (annual)	Annual htTKV change (%)

Quality Assessment

**Figure 2:**
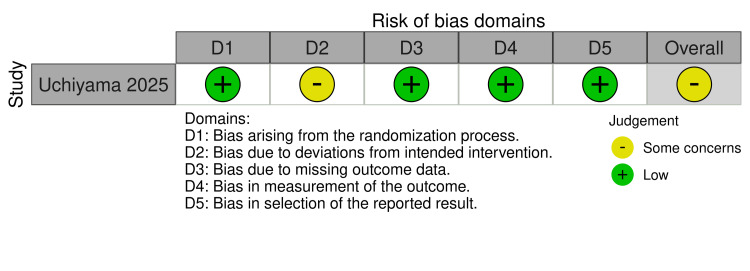
Quality assessment of the RCT (RoB2) [10] Applied to the only RCT included in this review. Rated across five RoB2 domains, rated as low risk, some concerns, or high risk. RCT = Randomized controlled trial; RoB2 = Risk of Bias 2

The single RCT assessed (Figure 2) using the RoB2 tool was rated with some concerns, primarily due to its open-label design and the absence of a washout period between crossover periods [10,13].

The two case series measured using JBI studies demonstrated good quality in all aspects, except for Yoshimoto et al. (2024) [23], which had unclearness in two domains regarding participant inclusion (Table 3). 

**Table 3:** Quality assessment of the cases series (JBI) [19,23] Applied to both case series that have no comparator group. JBI: Joanna Briggs Institute

Study ID	1-Were there clear criteria for inclusion in the case series?	2-Was the condition measured in a standard, reliable way for all participants included in the case series?	3-Were valid methods used for identification of the condition for all participants included in the case series?	4-Did the case series have consecutive inclusion of participants?	5- Did the case series have complete inclusion of participants?	6-Was there clear reporting of the demographics of the participants in the study?	7-Was there clear reporting of clinical information of the participants?	8-Were the outcomes or follow up results of cases clearly reported?	9-Was there clear reporting of the presenting site(s)/clinic(s) demographic information?	10-Was statistical analysis appropriate?
Morioka 2023 [19]	Yes	Yes	Yes	Yes	Yes	Yes	Yes	Yes	Yes	Yes
Yoshimoto 2024 [23]	Yes	Yes	Yes	Unclear	Unclear	Yes	Yes	Yes	Yes	Yes

The observational studies were assessed using the NOS, yielding three studies of good quality and two of poor quality (Table 4).

**Table 4:** Quality assessment of observational studies (NOS) [17,18,20-22] Applied to the five observational cohort studies. D1: Is the case definition adequate/representative of the exposed cohort? D2: Representative of the cases/selection of the non-exposed cohort. D3: Selection of controls/ascertainment of exposure. D4: Definition of controls/demonstration that outcome of interest was not present at start of study. D5: Ascertainment of exposure/assessment of outcome. D6: Same method of ascertainment for cases and controls/was follow-up long enough for outcomes to occur? D7: Non-response rate/adequacy of follow-up of cohorts. * = One star awarded; ** = Two stars awarded (comparability domain). Maximum score = 9 stars. NOS = Newcastle-Ottawa Scale

Study ID	Selection				Comparability	Outcome			Quality score
	D1	D2	D3	D4		D5	D6	D7	
Cau 2025 [17]	*		*	*	–	*	*		Poor
Eswarappa 2025 [18]	*	*	*	*	**	*	*	*	Good
Nakajima 2025 [20]	*	*	*	*	**	*	*		Good
Nishida 2025 [21]	*	*	*	*	–	*	*	*	Poor
Yen 2025 [22]	*	*	*	*	**	*	*	*	Good

Double Arm Outcomes

**Figure 3:**
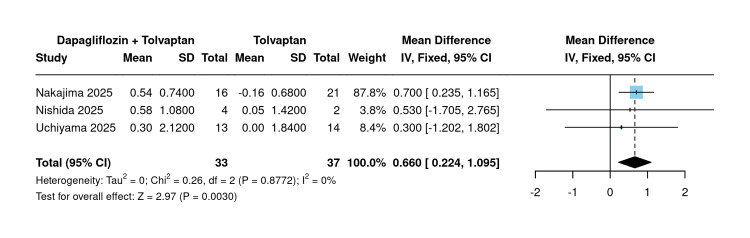
Mean change in hemoglobin [10,20,21] Forest plot of pooled mean difference in hemoglobin (g/dL) between dapagliflozin plus tolvaptan versus tolvaptan monotherapy across three studies.

Change in hemoglobin:* *Three studies comparing dapagliflozin added to tolvaptan versus tolvaptan monotherapy reported changes in hemoglobin. The pooled analysis using a fixed-effects model demonstrated a statistically significant increase in hemoglobin in favor of the dapagliflozin combination group, with a pooled MD of 0.66 g/dL (95% CI: 0.22 to 1.09; P=0.003) and no heterogeneity detected across studies (I²=0%; P=0.877) (Figure 3).

**Figure 4:**
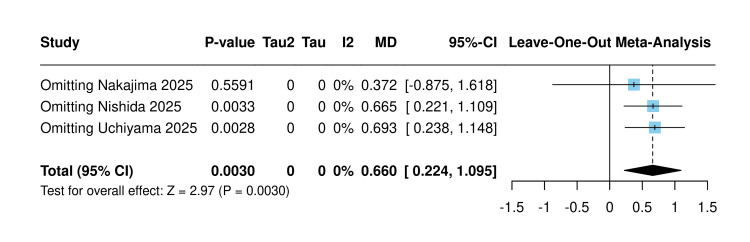
Sensitivity analysis mean change in hemoglobin [10,20,21]

Sensitivity analysis using the leave-one-out approach confirmed the robustness of this finding when omitting Nishida et al. (2025) (MD=0.66) and Uchiyama et al. (2025) (MD=0.69) [10,21]. However, the omission of Nakajima et al. (2025) rendered the result non-significant (MD=0.37; P=0.55) [20] (Figure 4).

**Figure 5:**
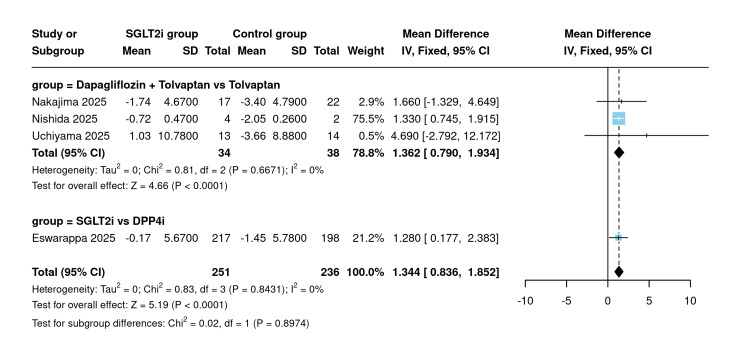
Post-eGFR slope [10,18,20,21] Forest plot of pooled mean difference in post-treatment eGFR slope (mL/min/1.73 m²/year) across four comparative studies stratified by comparator type. eGFR: Estimated glomerular filtration rate

Post-eGFR slope: Four studies reported post-treatment eGFR slope as a comparative outcome (n=251 SGLT2 inhibitor vs. 236 control). The analysis was stratified into two subgroups based on comparator type. In the dapagliflozin plus tolvaptan versus tolvaptan monotherapy subgroup, SGLT2 inhibitor use was associated with a significant attenuation of eGFR decline, with a pooled MD of 1.362 mL/min/1.73 m²/year (95% CI: 0.790 to 1.934; P<0.0001). In the SGLT2 inhibitor versus DPP4 inhibitor subgroup, a similar significant benefit was observed (MD=1.280; 95% CI: 0.177 to 2.383). The overall pooled estimate across both subgroups was 1.344 mL/min/1.73 m²/year (95% CI: 0.836 to 1.852; P<0.0001; I²=0%) (Figure 5).

**Figure 6:**
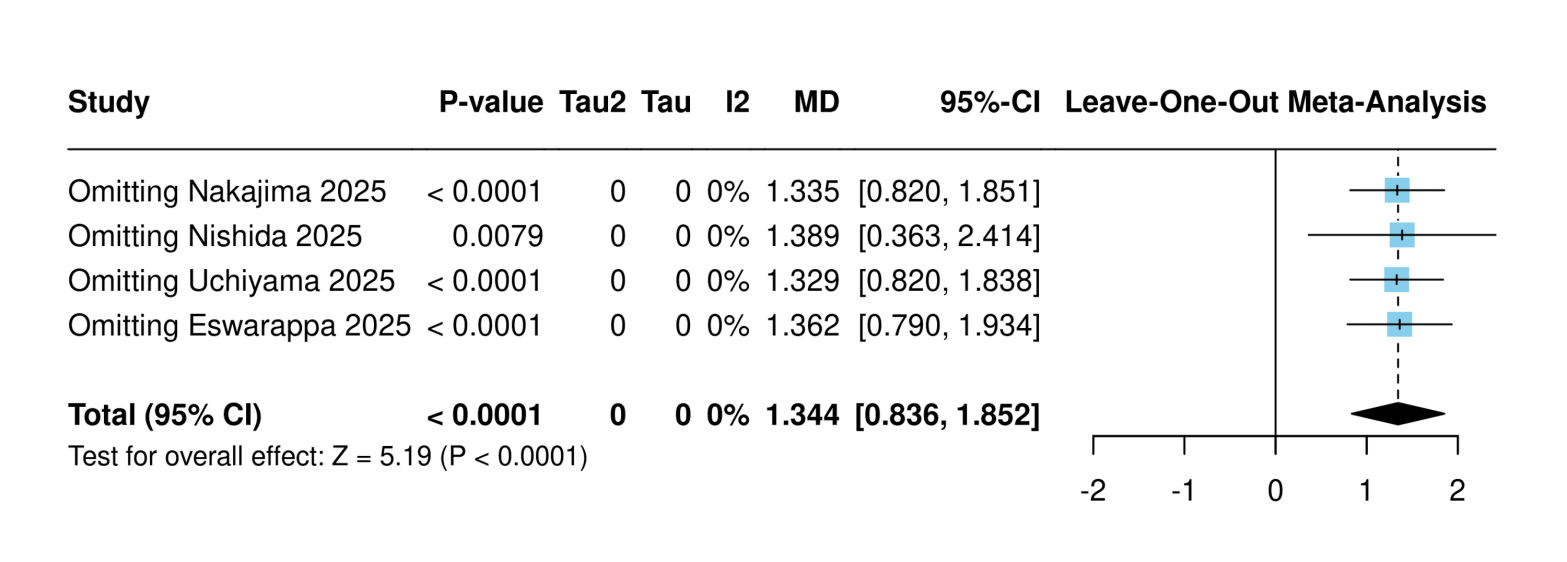
Sensitivity analysis of post-eGFR slope [10,18,20,21] eGFR: Estimated glomerular filtration rate

Sensitivity analysis using the leave-one-out method was made, and the results remained statistically significant regardless of which study was omitted (Figure 6).

Single Arm Outcomes

**Figure 7:**
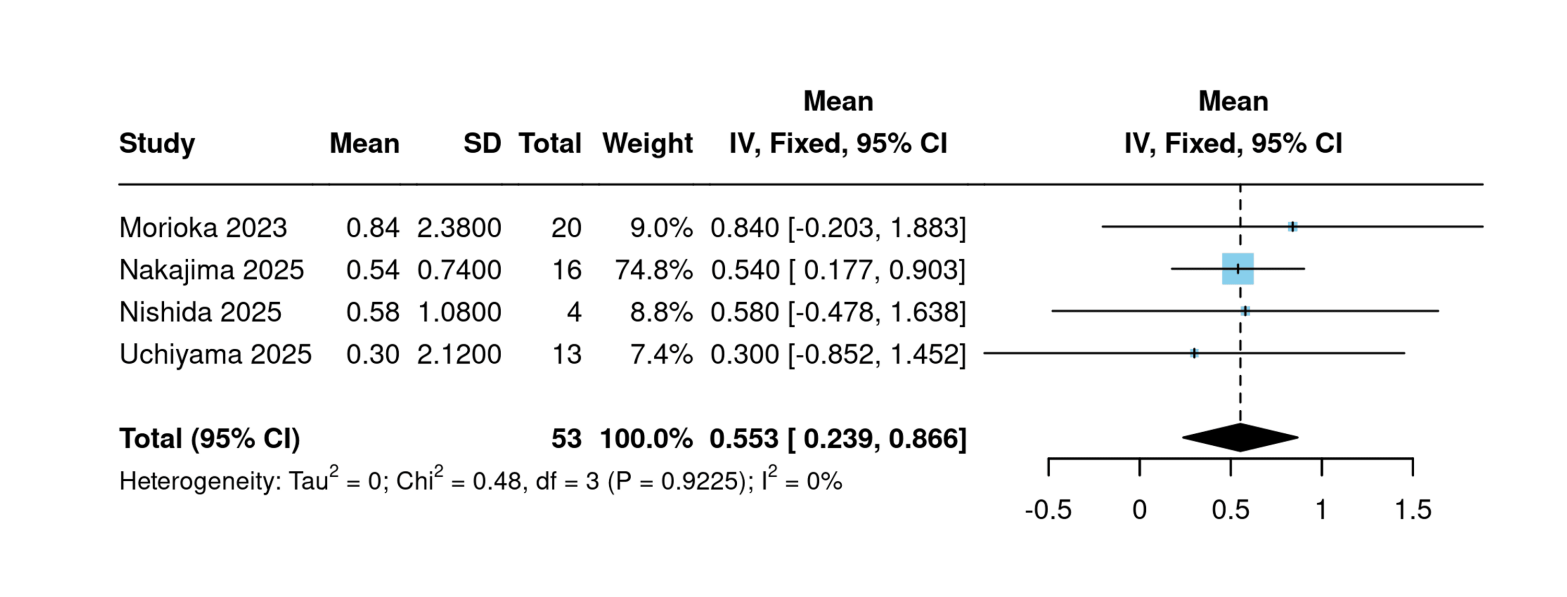
Mean change in hemoglobin (single arm) [10,19-21] Forest plot of pooled mean raw hemoglobin change (g/dL) across four single-arm studies following SGLT2 inhibitor initiation.

Change in hemoglobin: Four single-arm studies reported changes in hemoglobin following initiation of SGLT2 inhibitors. Using a fixed-effect model, the pooled mean raw change in hemoglobin was 0.55 g/dL (95% CI: 0.24 to 0.87; I²=0%; P=0.92), indicating a consistent and statistically significant increase in hemoglobin following SGLT2 inhibitor initiation across all included studies (Figure 7).

**Figure 8:**
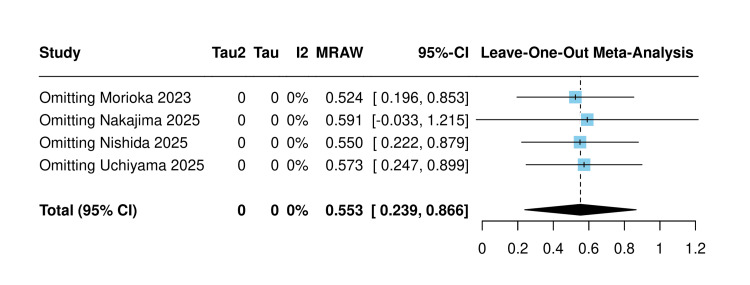
Sensitivity analysis of mean change in hemoglobin (single arm) [10,19-21]

Sensitivity analysis using the leave-one-out approach confirmed the stability of this estimate across all studies. Notably, omission of Nakajima et al. (2025) was the only scenario that rendered the lower confidence interval boundary marginally below zero, reflecting its substantial contribution to the pooled estimate given its dominant weight of 74.8% [20] (Figure 8).

**Figure 9:**
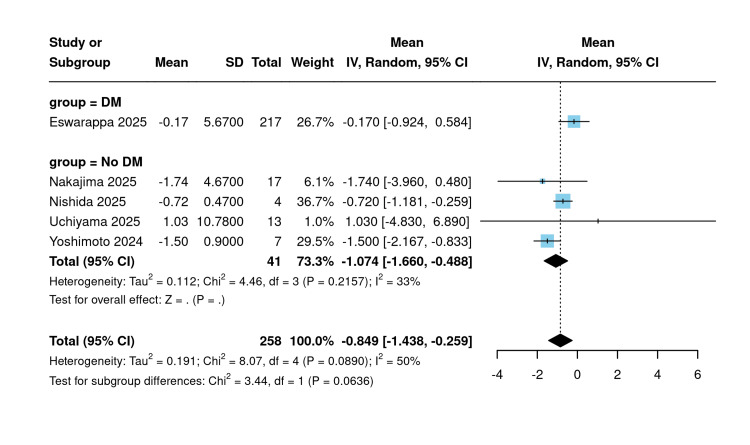
Post-eGFR slope subgrouped by diabetes [10,18,21,21,23] Forest plot stratifying single-arm post-treatment eGFR slope by diabetes mellitus status. eGFR: Estimated glomerular filtration rate

Post-eGFR slope: In the subgroup analysis by diabetes mellitus status, the DM subgroup (n=217) had a mean eGFR slope of −0.17 mL/min/1.73 m²/year (95% CI: −0.92 to 0.58), whereas the non-DM subgroup (n=41) demonstrated a greater decline, with a pooled mean of −1.07 mL/min/1.73 m²/year (95% CI: −1.66 to −0.49; I²=33%) (Figure 9).

**Figure 10:**
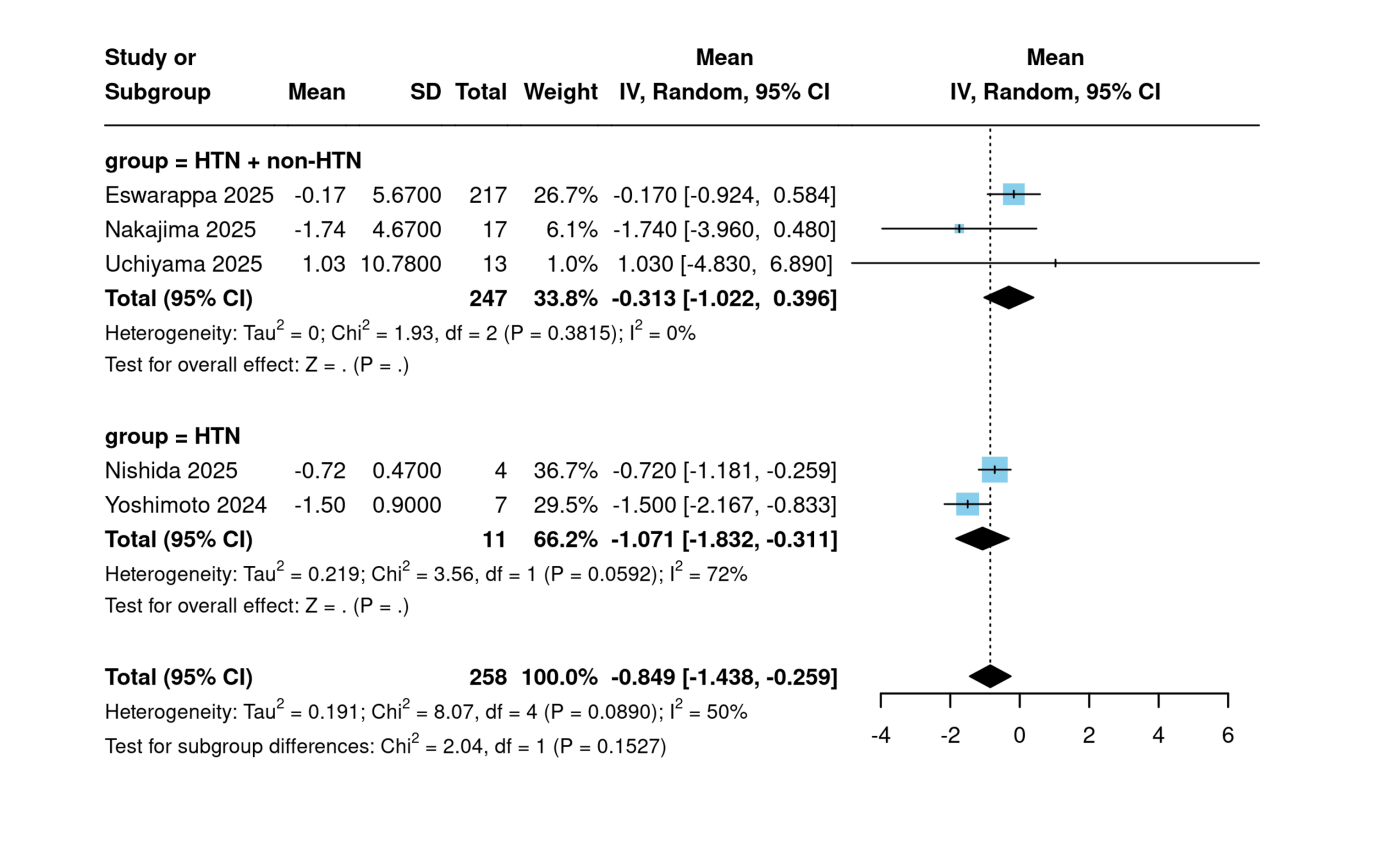
Post-eGFR slope subgrouped by hypertension [10,18,21,21,23] Forest plot stratifying single-arm post-treatment eGFR slope by hypertension status. eGFR: Estimated glomerular filtration rate

In the subgroup analysis by hypertension status, studies exclusively enrolling hypertensive patients (n=11) yielded a significant pooled mean eGFR slope of −1.07 mL/min/1.73 m²/year (95% CI: −1.83 to −0.31; I²=72%), whereas the mixed HTN/non-HTN subgroup (n=247) produced an estimate of −0.31 mL/min/1.73 m²/year (95% CI: −1.02 to 0.39; I²=0%). The subgroup difference was not statistically significant (χ²=2.04; p=0.153) (Figure 10).

**Figure 11:**
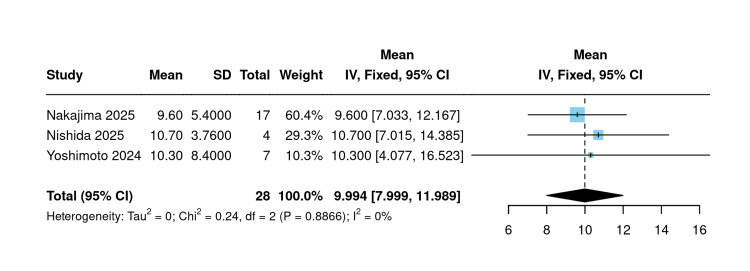
Percentage of initial dip in eGFR [20,21,23] Forest plot of pooled mean percentage initial eGFR dip across three single-arm studies. eGFR: Estimated glomerular filtration rate

Percentage initial eGFR dip (single-arm): Three studies reported the percentage initial eGFR dip following SGLT2 inhibitor initiation (n=28 total). Using a fixed-effects model, the pooled mean percentage initial eGFR dip was 9.99% (95% CI: 7.99 to 11.98; I²=0%; P=0.88), with no heterogeneity detected across studies (Figure 11).

**Figure 12:**
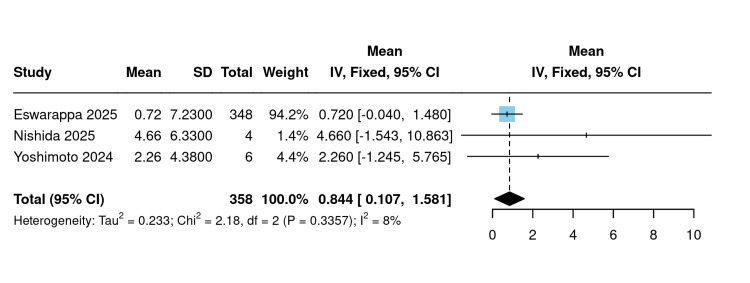
ΔeGFR slope [18,21,23] Forest plot of the pooled change in eGFR slope before versus after SGLT2 inhibitor initiation. eGFR: Estimated glomerular filtration rate

ΔeGFR slope (single-arm): Three studies reported the change in eGFR slope following SGLT2 inhibitor initiation (n=358 total). Using a fixed-effect model, the pooled mean ΔeGFR slope was 0.84 mL/min/1.73 m²/year (95% CI: 0.10 to 1.58; I²=8%; P=0.336). Heterogeneity was low across all studies (Figure 12).

Discussion

This systematic review and meta-analysis represent the first quantitative synthesis of evidence evaluating SGLT2 inhibitors in patients with ADPKD. The data were pooled from eight studies encompassing 3,180 patients. We demonstrate that SGLT2 inhibitor therapy is associated with a clinically meaningful and statistically significant attenuation of eGFR decline. Additionally, a consistent hemoglobin benefit was observed across both comparative and single-arm analyses. The pooled initial eGFR dip of approximaly 10% following SGLT2 inhibitor initiation, which confirms the well-established hemodynamic feedback response documented in DAPA-CKD, representing a pharmacodynamic marker of efficacy rather than nephron loss [4].

The attenuation of eGFR decline represents the primary and most clinically consequential finding of this analysis. The impact of background tolvaptan therapy has not been specifically examined in the literature; however, prior evidence from the TEMPO 3:4 and REPRISE trials demonstrates that tolvaptan significantly slows eGFR decline in ADPKD, and thus its use may have confounded the observed renal outcomes [2,24]. These results were consistent across both comparator types, whether dapagliflozin plus tolvaptan or tolvaptan monotherapy. Our pooled estimate harmonizes these individually underpowered observations into a robust, statistically significant signal, reinforcing the conclusion that the renoprotective effect of SGLT2 inhibitors extends to the ADPKD population.

Preclinical studies demonstrated that dual SGLT1/2 inhibition with phlorizin reduced cyst growth in Han: SPRD rats, whereas selective SGLT2 inhibition with dapagliflozin did not [25,26]. This suggests that compensatory glucose reabsorption via SGLT1 may promote cystogenesis when SGLT2 alone is inhibited. Systemically, SGLT2 inhibitor-induced osmotic diuresis stimulates vasopressin secretion, activating V2 receptor-cAMP signaling and driving cystic epithelial proliferation [27]. A modest increase in copeptin under euglycemic conditions provides a clinical correlate for this pathway [6]. Notably, the osmotic diuresis argument is weakened by the fact that tolvaptan, which induces far greater diuresis volumes, remains renoprotective in ADPKD [6].

A consistent and statistically significant increase in hemoglobin was observed across both comparative and single-arm analyses. This erythropoietic effect is a recognized class property of SGLT2 inhibitors, mediated by activation of the hypoxia-inducible factor pathway, which stimulates endogenous erythropoietin production [28]. In ADPKD, where anemia progressively develops as kidney function declines, this benefit carries additional clinical value beyond its established contribution to cardiovascular risk reduction [29]. Furthermore, SGLT2 inhibitor-induced ketogenesis has been shown to suppress cyst growth in rodent ADPKD models by curtailing the preferential glycolytic activity of cystic epithelial cells [30]. This suggests a potential disease-specific metabolic benefit that warrants prospective evaluation.

Morioka et al. (2023) observed that eGFR continued to decline beyond the initial dip rather than stabilizing, which they attributed to dapagliflozin-driven cyst enlargement [19]. In contrast, a large target trial emulation study reported post-dip eGFR stabilization at three to 12 months, mirroring non-ADPKD CKD behavior [17], with the discrepancy likely attributable to differences in follow-up duration, diabetes prevalence, and background tolvaptan use.

This meta-analysis has several strengths. It is the first quantitative synthesis dedicated to SGLT2 inhibitors in ADPKD, with pre-specified outcomes, subgroup analyses, and sensitivity analyses. However, important limitations must be acknowledged. Geographic distribution, which was heavily based on Japanese cohorts, limited external validity. Subgroup analyses by DM and HTN status were exploratory and underpowered. Follow-up durations varied widely, and the htTKV data were largely unavailable, precluding assessment of structural disease modification. All subgroup analyses were pre-specified but should be regarded as exploratory and hypothesis-generating, owing to the limited number of included studies and patients in each subgroup. Safety data, including urinary tract infection rates and CKD-mineral bone disorder markers, were incompletely reported across studies, despite the known risk of cyst infection accelerating ADPKD progression. Future studies should incorporate the htTKV and eGFR slope as co-primary endpoints, include genetic stratification, and systematically evaluate safety in combination with tolvaptan.

## Conclusions

Our findings demonstrate that SGLT2 inhibitor therapy in ADPKD is associated with a statistically significant attenuation of eGFR decline and a consistent increase in hemoglobin, with an initial eGFR dip representing a pharmacodynamic class effect rather than harm. These findings are predominantly derived from observational studies and should be regarded as hypothesis-generating rather than practice-changing; they do not constitute sufficient evidence to endorse routine SGLT2 inhibitor use in ADPKD outside of clinical trials. They do, however, provide a compelling rationale for prospective evaluation in adequately powered randomized trials with htTKV and eGFR slope as co-primary endpoints.

## Appendices

**Table 5:** Full detailed advanced search strategy

Database	Search query	Results
PubMed	("Polycystic kidney*" OR "Polycystic renal" OR "PKD" OR "ADPKD") AND (SGLT2 OR SGLT-2 OR "Sodium-glucose cotransporter 2 inhibitor*" OR "sodium-glucose transporter 2 inhibitor*" OR "Sodium Glucose Transporter 2 Inhibitor" OR SGLT2i OR gliflozin* OR dapagliflozin OR empagliflozin OR canagliflozin OR ertugliflozin OR ipragliflozin OR luseogliflozin OR tofogliflozin OR remogliflozin OR sergliflozin OR licogliflozin OR sotagliflozin OR bexagliflozin OR enavogliflozin OR rongliflozin)	44
Web of Science (WOS)	TS=(("Polycystic kidney*" OR "Polycystic renal" OR "PKD" OR "ADPKD") AND (SGLT2 OR SGLT-2 OR "Sodium-glucose cotransporter 2 inhibitor*" OR "sodium-glucose transporter 2 inhibitor*" OR "Sodium Glucose Transporter 2 Inhibitor" OR SGLT2i OR gliflozin* OR dapagliflozin OR empagliflozin OR canagliflozin OR ertugliflozin OR ipragliflozin OR luseogliflozin OR tofogliflozin OR remogliflozin OR sergliflozin OR licogliflozin OR sotagliflozin OR bexagliflozin OR enavogliflozin OR rongliflozin))	46
Scopus	TITLE-ABS-KEY(("Polycystic kidney*" OR "Polycystic renal" OR "PKD" OR "ADPKD") AND (SGLT2 OR SGLT-2 OR "Sodium-glucose cotransporter 2 inhibitor*" OR "sodium-glucose transporter 2 inhibitor*" OR "Sodium Glucose Transporter 2 Inhibitor" OR SGLT2i OR gliflozin* OR dapagliflozin OR empagliflozin OR canagliflozin OR ertugliflozin OR ipragliflozin OR luseogliflozin OR tofogliflozin OR remogliflozin OR sergliflozin OR licogliflozin OR sotagliflozin OR bexagliflozin OR enavogliflozin OR rongliflozin))	60
Cochrane Library	(("Polycystic kidney*" OR "Polycystic renal" OR "PKD" OR "ADPKD") AND (SGLT2 OR SGLT-2 OR "Sodium-glucose cotransporter 2 inhibitor*" OR "sodium-glucose transporter 2 inhibitor*" OR "Sodium Glucose Transporter 2 Inhibitor" OR SGLT2i OR gliflozin* OR dapagliflozin OR empagliflozin OR canagliflozin OR ertugliflozin OR ipragliflozin OR luseogliflozin OR tofogliflozin OR remogliflozin OR sergliflozin OR licogliflozin OR sotagliflozin OR bexagliflozin OR enavogliflozin OR rongliflozin)):ti,ab,kw	12
Embase	('polycystic kidney*':ti,ab,kw OR 'polycystic renal':ti,ab,kw OR 'pkd':ti,ab,kw OR 'adpkd':ti,ab,kw) AND (sglt2:ti,ab,kw OR 'sglt 2':ti,ab,kw OR 'sodium-glucose cotransporter 2 inhibitor*':ti,ab,kw OR 'sodium-glucose transporter 2 inhibitor*':ti,ab,kw OR 'sodium glucose transporter 2 inhibitor':ti,ab,kw OR sglt2i:ti,ab,kw OR gliflozin*:ti,ab,kw OR dapagliflozin:ti,ab,kw OR empagliflozin:ti,ab,kw OR canagliflozin:ti,ab,kw OR ertugliflozin:ti,ab,kw OR ipragliflozin:ti,ab,kw OR luseogliflozin:ti,ab,kw OR tofogliflozin:ti,ab,kw OR remogliflozin:ti,ab,kw OR sergliflozin:ti,ab,kw OR licogliflozin:ti,ab,kw OR sotagliflozin:ti,ab,kw OR bexagliflozin:ti,ab,kw OR enavogliflozin:ti,ab,kw OR rongliflozin:ti,ab,kw)	71
